# The Role of Diet Compared to Physical Activity on Women's Cancer Mortality: Results From the Third National Health and Nutrition Examination Survey

**DOI:** 10.3389/fpubh.2022.853636

**Published:** 2022-08-01

**Authors:** Joshua E. Chan, Michelle Ann Caesar, Amandeep K. Mann, Alex Koh-Bell, Michael T. Richardson, Caitlin R. Johnson, Daniel S. Kapp, John K. Chan

**Affiliations:** ^1^Department of Microbiology and Immunology, Stanford University School of Medicine, Stanford, CA, United States; ^2^Department of Obstetrics and Gynecology, California Pacific Medical Center Research Institute, San Francisco, CA, United States; ^3^Palo Alto Medical Foundation Research Institute, Palo Alto, CA, United States; ^4^California Pacific Medical Center, San Francisco, CA, United States; ^5^Department of Obstetrics and Gynecology, University of California, Los Angeles, Los Angeles, CA, United States; ^6^Department of Radiation Oncology, Stanford University School of Medicine, Stanford, CA, United States

**Keywords:** cancer, physical activity, diet, obesity, epidemiology, risk factors

## Abstract

**Background:**

Among women in the United States, cancer is the second leading cause of death. Prior studies have examined how lifestyle factors, such as diet and physical activity, influence cancer mortality. However, few have evaluated if diet or physical activity has a stronger protective effect for cancer mortality. Therefore, this study aims to evaluate and compare the impacts of diet and physical activity on women's cancer mortality.

**Methods:**

Prospective, cross-sectional data were abstracted from the Third US National Health and Nutrition Examination Survey (NHANES III) on female respondents from 1988 to 1994. Physical activity was derived from the CDC's metabolic equivalent (MET) intensity levels. Dietary classifications were derived from the USDA's healthy eating index (HEI). We utilized the National Death Index to obtain mortality follow-up information on our cohort until December 31, 2015. Chi-squared, multivariable Cox regression, and Kaplan–Meier estimates were employed for statistical analyses.

**Results:**

Of 3,590 women (median age: 57, range: 40–89), 30% had an obese BMI (BMI≥30 kg/m^2^). Additionally, 22% of participants self-reported a healthy diet, 69% needed dietary improvement, and 9% had a poor diet. Furthermore, 21% reported physical inactivity, 44% did not meet physical activity guidelines, and 35% met guidelines. On multivariate analysis, healthy diet (HR: 0.70; 95% CI: 0.51–0.98; *p* = 0.04), but not physical activity (HR: 0.87; 95% CI: 0.55–1.38; *p* = 0.55), independently predicted for lower cancer mortality. Participants with a healthy diet but low exercise had decreased cancer mortality compared to participants with an unhealthy diet but high exercise (*p* = 0.01).

**Conclusions:**

A healthful diet was associated with lower cancer mortality in women, even after adjusting for obesity, inflammation, and other covariates. In addition, diet may play a stronger role in reducing cancer mortality in women than physical activity.

## Introduction

Cancer is a leading cause of death for American women (1). A higher risk of disease has been attributed to a less healthful diet and sedentary lifestyle (2, 3). Furthermore, the benefits of a healthy diet and frequent exercise on health and longevity are well-established (4–8).

Multiple studies have reported protective effects of healthful diets on cancer risk and mortality (9–11). Diet has been associated with lower pancreatic cancer risk; however, this association is stronger in men when compared to women (9). A high intake of fruits, vegetables, and whole grains has been associated with lower risks for several cancers and overall cancer mortality (10).

The association between diet and cancer mortality has previously been studied among women, revealing that a healthful diet is protective against cancer (12). One prior study showed a 70% decrease in cancer risk associated with a healthful diet (13). While single foods like flax seeds and broccoli sprouts are shown to help prevent cancer, they do not accurately represent the overall healthfulness of one's diet; examining overall diet may therefore provide more accurate insights when examining cancer prevention and mortality (14, 15). For example, one clinical trial reported that a low-fat diet reduced breast cancer mortality (12). Similarly, another study reported that Chinese women who consume traditional diets rich in vegetables, fruit, soy, milk, poultry, and fish had a 74% reduction in breast cancer risk compared to women who regularly consume refined grain, meat, and pickles (16). Mediterranean diets rich in fruits, vegetables, legumes, and fish with smaller amounts of dairy and meat were also associated with decreased cancer risk and lower overall mortality (17). On the other hand, women with diets rich in refined flour, sugar, meat, and dairy had over twice the risk for developing colorectal cancer in comparison to women with Mediterranean diets (18). Insufficient nutrition may increase cancer risk through mechanisms of DNA damage, reduced tolerance toward endogenous and exogenous stressors, and impaired metabolism of carcinogens (19).

Physical activity has also been shown to decrease cancer incidence and mortality (20, 21). According to the World Cancer Research Fund, a sedentary lifestyle may alter metabolism to favor a tumorigenic cellular environment and impair immune and hormonal function (19). A review from Ruiz-Casado et al. found that consistent moderate-intensity physical activity decreased the risk of cancer and cancer mortality and described this protective effect in animal models (20). In a meta-analysis of over one million participants, Moore et al. found leisure-time physical activity to be associated with a lower risk for many types of cancers (21). Specifically among women, those who walked 3–5 h per week had the greatest risk reduction in breast cancer mortality compared to those who exercised less (22). Conversely, a study of over 15,000 individuals found leisure-time physical activity to be correlated with cardiovascular and respiratory disease mortality, though there was no association with cancer mortality (23).

Both poor diet and low physical activity have been associated with higher inflammation and obesity, two risk factors for cancer (24, 25). Higher baseline inflammation, measured by fibrinogen and C-reactive protein (CRP) levels, has been show to increase cancer risk and mortality (26, 27). A review of 93 studies on the protective effect of diet on cancer suggests that this impact may be due to antioxidant compounds in foods that decrease inflammation (11). In a review of the role of exercise in cancer prevention, Hojman describes how exercise can decrease both acute and chronic inflammation (28). The relationship between diet and exercise and obesity has also been established in prior reports, showing that both diet and exercise play a role in weight loss (29–31). Diet quality and exercise may be correlated, as particularly health-conscious individuals may have both a healthful diet and frequently exercise. While extensive research has been accomplished to determine the beneficial effects of a healthful diet on the reduction of cancer mortality, the combination of diet and exercise has not been extensively researched. Furthermore, cancer incidence and mortality rates are higher in men compared to women, which may suggest that gender modulates the role of lifestyle on cancer outcomes (32). Given these differences and the limitations of prior studies, we proposed to determine the isolated impacts of diet and exercise and their relative importance on women's cancer mortality through analysis of the Third National Health and Nutrition Examination Survey (NHANES III) dataset. Due to its large, nationally representative sample size and extensive use of interviews and physical examination to assess lifestyle habits, the NHANES III provided accurate data on trends and disparities in diet and exercise among US women.

This report aimed to identify the relationships between diet, exercise, and cancer mortality among women. We investigated the a priori hypothesis that diet has a greater protective effect on reducing cancer mortality compared to physical activity. Through multivariate analysis and stratified survival curves, we elucidated the relationships between diet, exercise, and cancer survival after adjusting for covariates related to demographic and clinical factors.

## Materials and Methods

### Data Source

This observational study abstracted prospective, cross-sectional data from the Third National Health and Nutrition Examination Survey (NHANES III) database from 1988 to 1994. This survey is a National Center for Health Statistics (NCHS) program conducted by the Centers for Disease Control and Prevention (CDC) (33). NHANES III conducted household interviews and physical examinations through mobile examination centers to assess the health and nutrition of a nationally representative sample of the US population. To ensure generalizability, the NHANES III used a multistage stratified, clustered probability sample and sampling weights. In addition, participants' data were linked to the National Death Index (NDI) through probabilistic matching using social security number, birth date, and other personal data to obtain cancer-specific mortality follow-up through December 31, 2015. The follow-up period for each of the NHANES III participants was calculated as either the time from the date of their home-interview for those 40 years or older until the occurrence of cancer death or the censor date, December 31, 2015. Total cancer mortality included deaths from all cancer sites (C00-C97) as defined by the 10th revision of the International Classification of Diseases (ICD-10). Participants' cancer subtype was not publicly available through the NDI database. Similar to prior studies examining the association between lifestyle factors and cancer mortality, we decided to limit our sample size to NHANES III participants to obtain extended cancer mortality follow-up (34, 35).

### Measure of Physical Activity

We followed the workflow established by Pate et al. and the CDC guidelines to categorize participants' physical activity levels (25). The Physical Activity Guidelines for Americans from the CDC recommends adults to have a minimum of 150 min of moderate-intensity aerobic activity, 75 min of vigorous-intensity activity, or an equivalent combination of moderate- and vigorous-intensity activity per week (36). The NHANES III assessed physical activity based on participants' self-reported frequency of engaging in walking, jogging, biking, swimming, dancing, calisthenics, yard work, lifting weights, and other forms of exercises, sports, or physically active hobbies over the past month. The frequency of physical activity was converted to weekly rates. In addition, intensity of activity was determined using metabolic equivalent (MET) levels defined in the NHANES database. Participants met recommended guidelines with moderate activity of 3–6 METs 5 times per week, vigorous activities of 6 or more METs 3 times per week, or an equivalent weighted combination of the two (37). Participants' activity levels were categorized into either meeting recommended guidelines, not meeting recommended guidelines, or inactive if they reported no physical activity.

### Measure of Diet

The Healthy Eating Index (HEI), developed by the United States Department of Agriculture (USDA) Center for Nutrition Policy and Promotion, was used to measure diet quality based on participants' food and nutrient intake (38). Unlike indices such as The Mediterranean Diet Score and Alternative Healthy Eating Index, the HEI assesses adherence to the US federal dietary guidelines (39, 40). High HEI adherence has previously been validated to predict for lower cancer mortality, which reduces the likelihood of effect modification between our primary exposure and outcome (41). Since NHANES III data ranges from 1988 to 1994, the most comparable HEI version during that time (1994–1996) was used (42). Dietary intake, calculated as participants' reported food intake in the past 24 h, was used to calculate HEI scores. The HEI is on a 100-point scale based on meeting recommendations for 10 components. Using the 1989–1996 version of the USDA Food Guide Pyramid guidelines, a full 10 points were given for meeting recommended servings in categories such as grains, fruits, vegetables, meats, and dairy, and any lesser intake was awarded points proportionally. The other four components consisted of total fat, saturated fat, cholesterol, and sodium, which were given a score of 10 if participants kept below recommended limits. The last component to calculate HEI scores was dietary variety. Based on USDA recommended guidelines and the scoring of 10 components, participants' HEI scores were categorized into good (HEI score>80), needs improvement (HEI score 51–80), and poor (HEI score <51) (42). According to USDA guidelines, a good dietary profile for a female 25–50 years of age was a minimum of 9 servings of grain, 4 servings of vegetables, 3 servings of fruit, 2 servings of milk, and 2.4 servings of meat ≤30% of kcal for total fat intake, <10% of kcal for saturated fat intake, ≤300 mg of cholesterol intake, ≤2,400 mg of sodium intake, and ≥8 item/day of food variety. For females 50 years and older, a good diet was classified as 7.4 servings of grain, 3.5 servings of vegetables, 2.5 servings of fruit, 2.0 servings of milk, and 2.2 servings of meat, ≤30% of kcal for total fat intake, <10% of kcal for saturated fat intake, ≤300 mg of cholesterol intake, ≤2,400 mg of sodium intake, and ≥8 item/day of food variety (28).

### Covariates

We included the following covariates in our analyses: age, race, education, income level, marital status, body mass index (BMI), smoking status, chronic health conditions, self-reported health status, and inflammatory biomarkers. Age was measured as a continuous variable. Participants were categorized as either White, Black, or Hispanic. Education level was dichotomized into at/below high school or above high school. Poverty-income ratio (PIR), the ratio of family income to the poverty threshold, was utilized to define income levels. The US Census defined income levels as poverty (ratio < 1), low income (1.0 ≤ ratio < 2.0), middle income (2.0 ≤ ratio < 4.0), and high income (ratio ≥4.0) (43). For simplification, we elected to combine poverty and low-income groups into the category of low income. Assessing participants' marital status, we categorized participants as single if they responded either that they were widowed, divorced, separated, or never married; whereas those who have responded of either being married or living with a partner were grouped as married or with a partner during their home-interview.

Participants' weight (kg) and standing height (cm) were measured in the mobile examination centers conducted by the CDC. These measurements were then used to calculate body mass index and converted to kg/m^2^. Participant BMI was classified as obese (≥30.0 kg/m^2^) or non-obese (<30.0 kg/m^2^). Smoking use was categorized into current, former, and never. NHANES participants reported their history of chronic health conditions (diagnosed by health professionals): arthritis, asthma, diabetes, emphysema, heart conditions, stroke, hip fracture, osteoporosis, spine fracture, wrist fracture, high blood pressure, and high cholesterol. Since participants could have multiple chronic health conditions, their reported conditions were summed and treated as continuous. During the home interview, participants were asked about their self-reported health status, “Would you say your health in general is excellent, very good, good, fair, or poor?” Participants who have responded excellent, very good, or good were grouped as “good” and those who responded fair or poor were grouped as “poor.” Since prior studies have shown an association between diet and physical activity with inflammation, we included CRP and fibrinogen as inflammatory biomarkers in our study (10, 44). Both inflammatory biomarkers were measured through analysis of blood samples.

### Inclusion Criteria

From the crude sample of 9,399 female participants who attended both household interviews and mobile examination centers, 9,176 women were eligible for mortality follow-up. Participants were considered eligible if the NDI had sufficient data to identify whether they were alive or deceased. We further limited our cohort to those who had complete information about their diet and physical activity (8,664). Since NHANES collected fibrinogen levels only from participants 40 years or older, our data only consisted of this age group (4,738). From this age group, we excluded women who reported being pregnant at the time of NHANES data collection because pregnant women have disparate activity levels compared to non-pregnant women (45). In addition, we excluded 554 participants who had a prior history of cancer. Six participants with CRP levels 10 mg/dL or above were not included in our cohort because abnormally high CRP levels are often due to active infection rather than chronic inflammation (46). Because NHANES III reported participants who are 90 years of age or above as “90,” we excluded 36 participants over 90 years old to obtain accurate age information. We excluded 190 participants reporting “other” race to understand how racial disparities in diet quality and physical activity influence cancer mortality. Women with missing information on CRP levels (216), education (20), poverty-income ratio (119), and BMI (7) were excluded, leaving a final sample size of 3,590 women.

### Statistical Analysis

All statistical analyses were performed using SAS Survey Proc procedures (e.g., SURVEYFREQ, SURVEYMEANS, and SRVEYPHREG) in which strata (SDPSTRA6), cluster (SDPPSU6), and weight (WTPFHX6) variables were used to accommodate the complex survey design of NHANES III. This ensured that no group was oversampled and that the results from the analyses would be representative of the US population. Due to the complexity of the survey data, we created a dummy variable as our analytical subset and used it in the domain of the survey procedure. These techniques were employed for all analyses to ensure that correct sample weights were utilized and reliable estimates were calculated.

We used Chi-square tests to measure the association of categorical factors in our study with physical activity levels and diet quality. Analysis of variance tests were used to compare the mean difference of CRP and fibrinogen levels between the subgroups of physical activity levels and diet quality. Kaplan–Meier survival curves were employed to evaluate the difference in cancer mortality among various combinations of diet quality and physical activity levels. We grouped participants as “Good diet, high physical activity” (HEI score>80 and meeting physical activity guidelines), “Good diet, low physical activity” (HEI score>80 and not meeting physical activity guidelines), “Poor diet, low physical activity” (HEI score<51 and not meeting physical activity guidelines), and “Poor diet, high physical activity” (HEI score<51 and meeting physical activity guidelines). Age was used as a baseline for time to follow-up for cancer death and was therefore excluded in the final model (47). We used Cox-proportional hazard models to analyze the impact of various factors on cancer mortality, adjusted for potential confounders. Confounding variables were determined based on their significant associations found in either the previous chi-square tests, ANOVA tests, or unadjusted cox-proportional hazard analyses. Proportional hazard assumptions were examined and met for all factors in the multivariate model. A *p*-value of <0.05 was considered statistically significant. Data analyses were conducted using SAS Enterprise Guide, version 7.1 (SAS Institute Inc., Cary, NC). This study was exempt from Institutional Review Board approval because we used a public-use data file with de-identified information of the participants in our study.

## Results

Baseline and clinical characteristics of the 3,590 participants identified in NHANES III (1988–1994) can be found in Table 1. The median age was 57 years (range: 40−9). White, black, and Hispanic participants comprised 85, 11, and 4% of our sample, respectively. The majority of participants had two chronic health conditions and 71% of the participants had a BMI of <30. Regarding smoking status, 54% of participants had never smoked, 26% were former smokers, and 21% participants were current smokers.

**Table 1:** Baseline characteristics of female participants by categories of physical activity and diet.

**Characteristics**	**Overall *N* = 3,590 (%)**	**Meeting physical activity criteria according to CDC^a^**				**Meeting dietary guidelines according to USDA^b^**			
		**Inactive (*N* = 1,060)**	**Not met (*N* = 1,493)**	**Met (*N* = 1,037)**	***P*-value**	**Poor (*N* = 257)**	**Needs improvement (*N* = 2,158)**	**Good (*N* = 781)**	***P*-value**
Age (years)									
Mean (range)	57 (40–89)	60 (40–89)	55 (40–89)	57 (40–89)	<0.001^c^	51 (40–86)	56 (40–89)	61 (40–89)	<0.001^c^
Race					<0.001^d^				<0.001^d^
White	85.1%	74.8%	86.6%	89.2%		80.5%	84.9%	87.5%	
Black	10.9%	18.8%	9.5%	8.0%		16.3%	11.3%	7.6%	
Hispanic	4.0%	6.4%	3.9%	2.8%		3.2%	3.8%	4.8%	
Education					<0.001^d^				0.12^d^
Below high school	27.0%	46.7%	25.6%	17.4%		28.0%	28.0%	23.5%	
High school	38.5%	34.6%	41.4%	37.1%		37.4%	39.3%	36.7%	
Above high school	34.40%	18.6%	32.9%	45.5%		34.6%	32.7%	39.8%	
Income level					<0.001^d^				0.99^d^
Low	36.7%	54.9%	33.6%	30.2%		36.9%	36.7%	37.0%	
Middle	35.2%	33.1%	36.3%	35.1%		36.0%	35.4%	34.4%	
High	28.0%	12.0%	30.1%	34.8%		27.1%	28.0%	28.6%	
Marital status					0.001^d^				0.6^d^
Single	36.7%	44.4%	33.0%	36.9%		33.6%	36.7%	38.0%	
Married or with partner	63.3%	55.6%	67.0%	63.1%		66.4%	63.3%	62.0%	
Mean chronic health conditions (range)	2 (1–9)	3 (1–9)	2 (1–9)	2 (1–9)	<0.001^c^	2 (1–7)	2 (1–9)	2 (1–9)	0.24^c^
Obesity					<0.001^d^				0.26^d^
<30 kg/m^2^	70.5%	61.7%	69.2%	77.2%		69.7%	69.6%	73.6%	
≥30 kg/m^2^	29.5%	38.3%	30.8%	22.8%		30.3%	30.4%	26.4%	
Self-reported health status					<0.001^d^				0.14^d^
Poor	20.3%	40.1%	17.0%	12.9%		21.8%	21.2%	17.1%	
Good	79.7%	59.9%	83.0%	87.1%		78.2%	78.8%	82.9%	
Smoking status					0.001^d^				<0.001^d^
Never	53.8%	46.3%	55.4%	56.1%		44.2%	52.4%	61.8%	
Former	25.5%	26.3%	23.2%	27.9%		26.8%	24.4%	28.2%	
Current	20.7%	27.4%	21.4%	16.0%		29.0%	23.1%	10.0%	
Inflammatory markers (mg/dL)									
Mean C-reactive protein (range)	0.51 (0.21–8.90)	0.66 (0.21–8.90)	0.49 (0.21–7.92)	0.44 (0.21–8.00)	<0.001^c^	0.57 (0.21–6.50)	0.51 (0.21–8.90)	0.49 (0.21–8.00)	0.45^c^
Mean fibrinogen (range)	308 (26–957)	334 (26–957)	305 (30–928)	297 (30–657)	<0.001^c^	297 (127–809)	310 (26–957)	306 (30–662)	0.11^c^
Meeting physical activity criteria^a^									<0.001^d^
Inactive	20.5%					26.8%	21.3%	15.3%	
Not Met	44.4%					46.3%	46.9%	35.9%	
Met	35.1%					26.9%	31.7%	48.9%	
Meeting dietary guidelines^b^					<0.001^d^				
Poor	8.7%	11.4%	9.1%	6.7%					
Needs improvement	69.1%	72.0%	73.0%	62.4%					
Good	22.2%	16.6%	18.0%	30.9%					

a *Meeting Center for Control Disease Guidelines for Physical Activity*.

b *Meeting US Department of Agriculture Food Guide Pyramid Recommended Guidelines for Healthy Diet*.

c *Analysis of Variance (ANOVA) tests were used to calculate p-value for these associations*.

d *Chi-square tests were used to calculate p-values for these associations*.

According to the standards set by the Centers for Disease Control Guidelines for Physical Activity, 35% of participants met the physical activity guidelines, 44% did not meet guidelines, and 21% were inactive. As defined by the US Department of Agriculture Food Guide Pyramid Recommended Guidelines for Healthy Diet, 22% were categorized as “good,” 69% as “needs improvement,” and 9% as “poor.” Participants were also asked to self-report their health status. Eighty percent of participants self-reported their health status as “good” compared to 20% who self-reported as “poor.”

The mean CRP level for all participants was 0.51 mg/dL (range = 0.21–8.9 mg/dL), and mean fibrinogen was 308 mg/dL (range = 26–957 mg/dL) (Table 1). Of note, there was no statistically significant association between diet and CRP (*p* = 0.45) or fibrinogen level (*p* = 0.11). However, CRP and fibrinogen levels were lower among more physically active participants. CRP levels were 0.66, 0.49, and 0.44 mg/dL among participants who were inactive, did not meet physical activity guidelines, and met physical activity guidelines, respectively (*p* < 0.001). Consistent with this trend, fibrinogen levels were 334, 305, and 297 mg/dL among inactive participants, those who did not meet activity guidelines, and those who met activity guidelines, respectively (*p* < 0.001).

**Figure 1:**
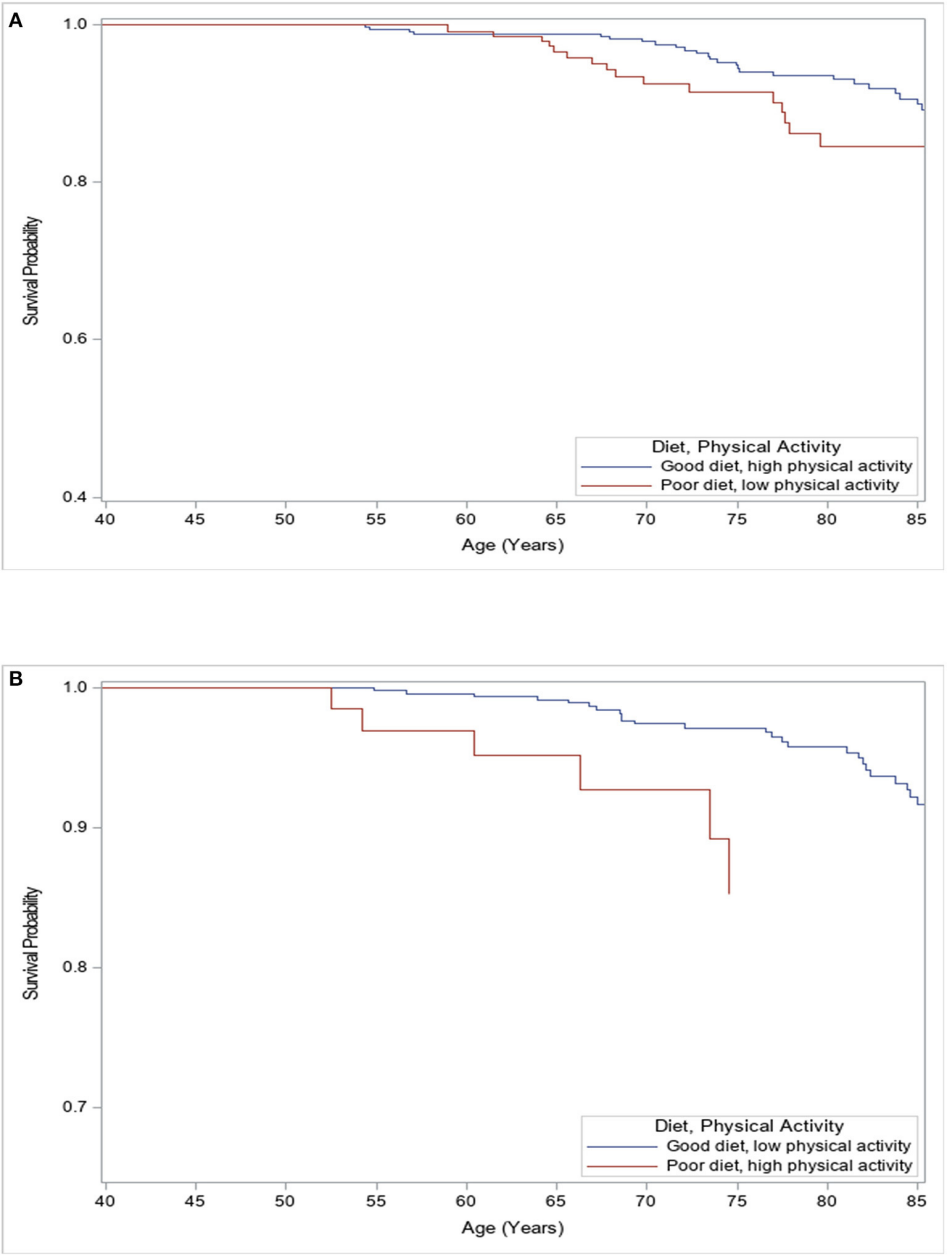
Kaplan–Meier analysis for freedom of all-cancer mortality among females according to levels of physical activity and quality of diet. **(A)** Female participants with poor diet and low physical activity levels had worse survival compared to those with good diet and high physical activity levels (Log-rank *p* = 0.09). **(B)** Female participants with a good diet and low physical activity levels had better survival compared to those with poor diet and high physical activity levels (Log-rank *p* = 0.01).

Those with a good diet and high physical activity had a survival advantage compared to those with a poor diet and low physical activity, though not statistically significant (*p* = 0.09, Figure 1A). Participants with a good diet and low physical activity had a significantly higher cancer survival compared to those with a poor diet and high physical activity (*p* = 0.01, Figure 1B).

The multivariate analysis was adjusted by confounding variables determined by their significant associations depicted in Tables 1, 2. On multivariate analysis, middle income (HR: 0.69; 95% CI: 0.48–0.99, *p* = 0.04), high income (HR: 0.64, 95% CI: 0.42–0.97, *p* = 0.04) and good diet (HR: 0.70, 95% CI: 0.51–0.98, *p* = 0.04) were independently associated with higher cancer survival (Table 3). However, women who were current smokers (HR: 3.72, 95% CI: 2.22–6.24, *p* < 0.001), formerly smoked (HR: 1.84, 95% CI: 1.05–3.21, *p* = 0.04), and were married or with a partner (HR: 1.46, 95% CI: 1.04–2.06, *p* = 0.03) had a lower cancer survival. Race, education, mean number of chronic health conditions, and CRP and fibrinogen levels were not associated with cancer survival (all *p* > 0.10). Of note, neither meeting physical activity criteria nor self-reported health status were independent factors for cancer survival (both *p* > 0.50).

**Table 2:** Unadjusted Hazard Ratios (HR) with 95% Confidence Intervals (95%CI) for female all-cancer mortality according to the main predictors (and other covariates).

**Characteristics**	**Hazard ratio**	**95% Confidence interval**	***P*-value**
Race
White	1		
Black	1.17	0.77–1.77	0.46
Hispanic	0.92	0.63–1.34	0.65
Education
Below high school	1		
High school	1.01	0.72–1.40	0.97
Above high school	1.11	0.70–1.77	0.65
Income level
Low	1		
Middle	0.77	0.52–1.14	0.18
High	0.81	0.56–1.16	0.24
Marital status
Single	1		
Married or with partner	1.21	0.87–1.68	0.26
Chronic health conditions	0.98	0.86–1.11	0.71
Obesity
<30 kg/m^2^	1		
≥30 kg/m^2^	1.46	1.00–2.13	0.048
Meeting physical activity criteria^a^
Inactive	1		
Not met	1.07	0.73–1.56	0.74
Met	0.75	0.48–1.17	0.20
Meeting dietary guidelines^b^
Needs improvement	1		
Good	0.61	0.43–0.87	0.01
Poor	1.59	0.78–3.21	0.20
Self-reported health status
Poor	1		
Good	1.05	0.68–1.61	0.84
Smoking status
Never	1		
Former	1.79	1.04–3.09	0.04
Current	3.73	2.26–6.15	<0.001
C-reactive protein	1.12	0.93–1.34	0.22
Fibrinogen	1.001	0.999–1.003	0.27

a *Meeting Center for Control Disease Guidelines for Physical Activity*.

b *Meeting US Department of Agriculture Food Guide Pyramid Recommended Guidelines for Healthy Diet*.

**Table 3:** Adjusted Hazard Ratios (HR) with 95% Confidence Intervals (95%CI) for female all-cancer mortality according to the main predictors (and other covariates).

**Characteristics**	**Hazard ratio**	**95% Confidence interval**	***P*-value**
Race
White	1		
Black	1.04	0.69–1.58	0.84
Hispanic	0.98	0.66–1.46	0.93
Education
Below high school	1		
High school	1.07	0.74–1.54	0.71
Above high school	1.45	0.91–2.31	0.12
Income level
Low	1		
Middle	0.69	0.48–0.99	0.04
High	0.64	0.42–0.97	0.04
Marital status
Single	1		
Married or with partner	1.46	1.04–2.06	0.03
Chronic health conditions	0.97	0.86–1.09	0.58
Obesity			
<30 kg/m^2^	1		
≥30 kg/m^2^	1.48	0.98–2.24	0.06
Meeting physical activity criteria^a^
Inactive	1		
Not Met	1.15	0.82–1.61	0.42
Met	0.87	0.55–1.38	0.55
Meeting dietary guidelines^b^
Needs improvement	1		
Good	0.70	0.51–0.98	0.04
Poor	1.49	0.73–3.03	0.27
Self-reported health status
Poor	1		
Good	1.21	0.76–1.94	0.42
Smoking status
Never	1		
Former	1.84	1.05–3.21	0.04
Current	3.72	2.22–6.24	<0.001
C-reactive protein	1.04	0.82–1.32	0.76
Fibrinogen	1.001	0.998–1.003	0.56

a *Meeting Center for Control Disease Guidelines for Physical Activity*.

b *Meeting US Department of Agriculture Food Guide Pyramid Recommended Guidelines for Healthy Diet*.

## Discussion

The current study utilized the NHANES nutritional data and the NDI to assess the relationships among physical activity, diet quality, and cancer mortality. As NHANES is nationally representative of the US population, these results are generalizable to non-pregnant women aged 40–89 years residing in the United States with no prior cancer history.

Similar to previous studies, we reported a strong association between cancer mortality and dietary quality. A 2016 systematic review by Kohler et al. found improved cancer survival in women who were more adherent to established American Cancer Society guidelines on increased physical activity and improved diet (48, 49). We also found diet to be an independent predictor of cancer survival. Proposed mechanisms of improved cancer survival in those with a better diet include the prevention of abnormal angiogenesis, as demonstrated in previous meta-analyses (10).

However, our study did not demonstrate improved survival for those with improved physical activity. This finding may in part be explained by the fact that our cohort was composed of participants with a variety of cancer types due to the lack of cancer-specific data available from NHANES. Several US studies on breast (22) and colon cancer (50), two of the most common cancers among US women (51, 52), have found a survival benefit with greater physical activity (53). However, this survival benefit has not been found in other forms of cancer (54). Future analyses that limit their samples to participants with cancers more amenable to lifestyle changes may provide different insights on cancer survival. Increased physical activity has been shown to increase lymphocyte proliferation and natural killer cytotoxic activity (55) as well as reduce patient fatigue, resulting in better toleration of systemic therapy (56). Although these findings may suggest improved cancer survival, other studies utilizing the NHANES III database have also not shown a survival advantage in cancer with improved physical activity (23).

Additionally, previous papers have demonstrated the association of higher inflammatory markers with cancer mortality (27, 57–59). However, while higher CRP and fibrinogen levels were associated with lower physical activity, they were not independent predictors for cancer mortality in our study. One explanation for this may be that the changes in inflammatory biomarker levels due to exercise may not be large enough to impact cancer mortality. Another rationale may be that previous studies have found inflammatory markers to be independent predictors of mortality only in men, whereas studies exclusively in women report no such finding (27).

Of note for practitioners working with cancer patients was the finding that participants in our study reported a better diet than objective measures suggested. Specifically, 80% of study participants self-reported their health status as “good,” yet only 22% of participants in fact met the dietary guidelines as set by the US Department of Agriculture Food Guide Pyramid Recommended Guidelines for Healthy diet. Participants' overestimation of their own health has been studied in depth in the past (60–62). If participants overestimate their health status, then some individuals with poor health would have been incorrectly classified as having good health. This incorrect classification would worsen the outcomes of participants who truly are in good health. Therefore, the benefits of having a good health status may be greater than they appear in this study. Physicians and other practitioners caring for cancer participants should be aware of this patient misunderstanding and work to educate participants on what factors are associated with improved cancer survival. Clinical care for cancer patients should incorporate referrals to dieticians so patients can be better equipped to maintain a healthy diet during and after cancer treatment, as diets high in fiber and low in fat are associated with lower cancer progression and risk of recurrence (63).

## Strengths and Limitations

Our study is limited by information bias. For example, the NHANES database collects self-reported dietary data that may not accurately reflect participants' diet; as described previously, poor dietary choices are often underreported (64). Furthermore, the NHANES III only records participants' frequency of physical activity, but not duration of activity; therefore, it is unknown how different durations of physical activity impact cancer survival (65–68). Future studies could include biometric measurements (e.g., heart rate, blood oxygen concentration) during physical activity in addition to tracking total minutes and type of exercise. In addition, the cross-sectional nature of the NHANES III data only allows us to infer associations between clinical factors and cancer mortality rather than causation. Because NHANES measurements were only measured at one point in time, the effect of changes in risk factors over time on cancer mortality could not be determined. Finally, the NHANES and NDI databases do not provide publicly available information on participants' cancer type, stage of disease, time of diagnosis or surgical or systemic treatments. Neither are the genetic predispositions of participants known nor their cause of death. While the lack of data on cancer type prevents us from making specific recommendations on physical activity for cancer prevention, our results may provide general guidance on how lifestyle factors influence cancer survival. In addition, the strengths of our study include the robust nature of the NHANES database as a large, prospective, nationally representative database with extensive time periods evaluated. Through novel multivariate analysis and Kaplan–Meier survival curves, this study is among the first to find that diet may play a stronger role in reducing cancer mortality in women compared to physical activity.

## Conclusions

In conclusion, this comprehensive study from the NHANES database found a significant survival improvement in those participants with a healthy diet, but no difference in survival by physical activity level. Physicians and others who treat participants with cancer should be aware of this survival advantage and incorporate wellbeing and healthy living as a core component of their oncologic practice, with particular attention to patients' perception of their own health status. Further research should be conducted into the relationships among physical activity, diet, and cancer mortality, particularly given recent utilization of technological advancements which better allow for precise measurement of diet and physical activity.

## Data Availability Statement

Publicly available datasets were analyzed in this study. This data can be found here: https://www.cdc.gov/nchs/nhanes/nh3data.htm.

## Author Contributions

Conception and design: JosC, AM, AK-B, MR, DK, and JohC. Data acquisition, data analysis, and data interpretation: AM, MC, and CJ. Editing: JosC, MC, AM, CJ, AK-B, MR, DK, and JohC. All authors contributed to the article and approved the submitted version.

## Funding

This work was supported by the Denise Hale and Fisher Family Foundation. A version of this work was presented at the Society for Gynecologic Oncology 2019 conference.

## Conflict of Interest

JohC has received non-specific funding from Denise Cobb Hale Chair and the Fisher Family Fund; consulted for AstraZeneca, Glaxosmithkline, and Myriad; received payment or honoraria from AstraZeneca, Clovis, Eisai, Glaxosmithkline, Merck, and Roche; and participated on a Data Safety Monitoring Board or Advisory Board for AbbVie, AstraZeneca, Clovis, Eisai, Glaxosmithkline, Immunogen, Myriad, Roche, and Seagen. The remaining authors declare that the research was conducted in the absence of any commercial or financial relationships that could be construed as a potential conflict of interest.

## Publisher's Note

All claims expressed in this article are solely those of the authors and do not necessarily represent those of their affiliated organizations, or those of the publisher, the editors and the reviewers. Any product that may be evaluated in this article, or claim that may be made by its manufacturer, is not guaranteed or endorsed by the publisher.

## References

[1] CDC. Leading Causes of Death-Females-All races/origins. Centers for Disease Control and Prevention (2021). Available online at: https://www.cdc.gov/women/lcod/2017/all-races-origins/index.htm (accessed April 02, 2022).

[2] Kerr J Anderson C Lippman SM. Physical activity, sedentary behaviour, diet, and cancer: an update and emerging new evidence. Lancet Oncol. (2017) 18:e457–71. 10.1016/S1470-2045(17)30411-4 28759385 PMC10441558

[3] Pierce JP Stefanick ML Flatt SW Natarajan L Sternfeld B Madlensky L. Greater survival after breast cancer in physically active women with high vegetable-fruit intake regardless of obesity. JCO. (2007) 25:2345–51. 10.1200/JCO.2006.08.6819 17557947 PMC2274898

[4] Gremeaux V Gayda M Lepers R Sosner P Juneau M Nigam A. Exercise and longevity. Maturitas. (2012) 73:312–7. 10.1016/j.maturitas.2012.09.012 23063021

[5] Zhang FF Cudhea F Shan Z Michaud DS Imamura F Eom H. Preventable cancer burden associated with poor diet in the United States. JNCI Cancer Spectrum. (2019) 3:pkz034. 10.1093/jncics/pkz034 31360907 PMC6649723

[6] Tavakol Z Ghannadi S Tabesh MR Halabchi F Noormohammadpour P Akbarpour S. Relationship between physical activity, healthy lifestyle and COVID-19 disease severity; a cross-sectional study. J Public Health. (2021) 1–9. 10.1007/s10389-020-01468-9. [Epub ahead of print]. 33558839 PMC7858040

[7] Wang Q Zhou W. Roles and molecular mechanisms of physical exercise in cancer prevention and treatment. J Sport Health Sci. (2021) 10:201–10. 10.1016/j.jshs.2020.07.008 32738520 PMC7987556

[8] Millar SR Navarro P Harrington JM Perry IJ Phillips CM. Dietary quality determined by the healthy eating index-2015 and biomarkers of chronic low-grade inflammation: a cross-sectional analysis in middle-to-older aged adults. Nutrients. (2021) 13:222. 10.3390/nu13010222 33466696 PMC7828829

[9] Zheng J Guinter MA Merchant AT Wirth MD Zhang J Stolzenberg-Solomon RZ. Dietary patterns and risk of pancreatic cancer: a systematic review. Nutr Rev. (2017) 75:883–908. 10.1093/nutrit/nux038 29025004 PMC5914454

[10] Schwingshackl L Schwedhelm C Galbete C Hoffmann G. Adherence to mediterranean diet and risk of cancer: an updated systematic review and meta-analysis. Nutrients. (2017) 9:E1063. 10.3390/nu9101063 28954418 PMC5691680

[11] Grosso G Bella F Godos J Sciacca S Del Rio D Ray S. Possible role of diet in cancer: systematic review and multiple meta-analyses of dietary patterns, lifestyle factors, and cancer risk. Nutr Rev. (2017) 75:405–19. 10.1093/nutrit/nux012 28969358

[12] Chlebowski RT Anderson GL Manson JE Prentice RL Aragaki AK Snetselaar L. Low-fat dietary pattern and cancer mortality in the women's health initiative (WHI) randomized controlled trial. JNCI Cancer Spectrum. (2018) 2:pky065. 10.1093/jncics/pky065 31360880 PMC6649760

[13] Mw R Mw R. Scientific Evaluation of Dietary Factors in Cancer. Available online at: https://clinmedjournals.org/articles/jnmdc/journal-of-nutritional-medicine-and-diet-care-jnmdc-4-029.php?jid=jnmdc (accessed April 18, 2022).

[14] Ocké MC. Evaluation of methodologies for assessing the overall diet: dietary quality scores and dietary pattern analysis. Proc Nutr Soc. (2013) 72:191–9. 10.1017/S0029665113000013 23360896

[15] Donaldson MS. Nutrition and cancer: a review of the evidence for an anti-cancer diet. Nutr J. (2004) 3:19. 10.1186/1475-2891-3-19 15496224 PMC526387

[16] Zhang CX Ho SC Fu JH Cheng SZ Chen YM Lin FY. Dietary patterns and breast cancer risk among Chinese women. Cancer Causes Control. (2011) 22:115–24. 10.1007/s10552-010-9681-8 21080051

[17] Adherence to a Mediterranean Diet and Survival in a Greek Population. NEJM. Available online at: https://www.nejm.org/doi/10.1056/NEJMoa025039?url_ver=Z39.88-2003&rfr_id=ori%3Arid%3Acrossref.org&rfr_dat=cr_pub++0www.ncbi.nlm.nih.gov (accessed April 18, 2022).

[18] Mentella MC Scaldaferri F Ricci C Gasbarrini A Miggiano GAD. Cancer and mediterranean diet: a review. Nutrients. (2019) 11:2059. 10.3390/nu11092059 31480794 PMC6770822

[19] About the Third Expert Report. WCRF International. Available online at: https://www.wcrf.org/diet-activity-and-cancer/global-cancer-update-programme/about-the-third-expert-report/ (accessed April 18, 2022).

[20] Ruiz-Casado A Martín-Ruiz A Pérez LM Provencio M Fiuza-Luces C Lucia A. Exercise and the hallmarks of cancer. Trends Cancer. (2017) 3:423–41. 10.1016/j.trecan.2017.04.007 28718417

[21] Moore SC Lee IM Weiderpass E Campbell PT Sampson JN Kitahara CM. Association of leisure-time physical activity with risk of 26 types of cancer in 1.44 million adults. JAMA Intern Med. (2016) 176:816–25. 10.1001/jamainternmed.2016.1548 27183032 PMC5812009

[22] Holmes MD Chen WY Feskanich D Kroenke CH Colditz GA. Physical activity and survival after breast cancer diagnosis. JAMA. (2005) 293:2479–86. 10.1001/jama.293.20.2479 15914748

[23] El Saadany T Richard A Wanner M Rohrmann S. Sex-specific effects of leisure-time physical activity on cause-specific mortality in NHANES III. Prev Med. (2017) 101:53–9. 10.1016/j.ypmed.2017.05.029 28579493

[24] Ackerman SE Blackburn OA Marchildon F Cohen P. Insights into the link between obesity and cancer. Curr Obes Rep. (2017) 6:195–203. 10.1007/s13679-017-0263-x 28434109

[25] Font-Burgada J Sun B Karin M. Obesity and cancer: the oil that feeds the flame. Cell Metab. (2016) 23:48–62. 10.1016/j.cmet.2015.12.015 26771116

[26] Allin KH Bojesen SE Nordestgaard BG. Inflammatory biomarkers and risk of cancer in 84,000 individuals from the general population. Int J Cancer. (2016) 139:1493–500. 10.1002/ijc.30194 27194008

[27] Wulaningsih W Holmberg L Ng T Rohrmann S Van Hemelrijck M. Serum leptin, C-reactive protein, and cancer mortality in the NHANES III. Cancer Med. (2016) 5:120–8. 10.1002/cam4.570 26632325 PMC4708908

[28] Hojman P. Exercise protects from cancer through regulation of immune function and inflammation. Biochem Soc Trans. (2017) 45:905–11. 10.1042/BST20160466 28673937

[29] Allison RL. Back to basics: the effect of healthy diet and exercise on chronic disease management. S D Med. (2017) 10–18. 28817856

[30] Chin SH Kahathuduwa CN Binks M. Physical activity and obesity: what we know and what we need to know. Obes Rev. (2016) 17:1226–44. 10.1111/obr.12460 27743411

[31] Manore MM Larson-Meyer DE Lindsay AR Hongu N Houtkooper L. Dynamic energy balance: an integrated framework for discussing diet and physical activity in obesity prevention-is it more than eating less and exercising more? Nutrients. (2017) 9:E905. 10.3390/nu9080905 28825615 PMC5579698

[32] Siegel RL Miller KD Jemal A. Cancer Statistics, 2017. CA Cancer J Clin. (2017) 67:7–30. 10.3322/caac.21387 28055103

[33] U.S. Cancer Statistics Data Visualizations Tool. CDC (2021). Available online at: https://www.cdc.gov/cancer/uscs/dataviz/index.htm (accessed October 18, 2021).

[34] Richard A Martin B Wanner M Eichholzer M Rohrmann S. Effects of leisure-time and occupational physical activity on total mortality risk in NHANES III according to sex, ethnicity, central obesity, and age. J Phys Act Health. (2015) 12:184–92. 10.1123/jpah.2013-0198 24770336

[35] Deshmukh AA Shirvani SM Likhacheva A Chhatwal J Chiao EY Sonawane K. The association between dietary quality and overall and cancer-specific mortality among cancer survivors, NHANES III. JNCI Cancer Spectr. (2018) 2:pky022. 10.1093/jncics/pky022 29905226 PMC5989369

[36] CDC. Walking: The Physical Activity Guidelines for Americans. Centers for Disease Control and Prevention (2022). Available online at: https://www.cdc.gov/physicalactivity/walking/index.htm (accessed April 16, 2022).

[37] Beddhu S Baird BC Zitterkoph J Neilson J Greene T. Physical activity and mortality in chronic kidney disease (NHANES III). Clin J Am Soc Nephrol. (2009) 4:1901–6. 10.2215/CJN.01970309 19820134 PMC2798872

[38] Overview & Background of Healthy Eating Index (HEI). EGRP/DCCPS/NCI/NIH. Available online at: https://epi.grants.cancer.gov/hei/ (accessed April 3, 2022).

[39] Defining diet quality: a synthesis of dietary quality metrics and their validity for the double burden of malnutrition - The Lancet Planetary Health. Available online at: https://www.thelancet.com/journals/lanplh/article/PIIS2542-5196(20)30162-5/fulltext (accessed April 18, 2022). 10.1016/S2542-5196(20)30162-5 PMC7435701 32800153

[40] Healthy Eating Index. Food and Nutrition Service. Available online at: https://www.fns.usda.gov/healthy-eating-index-hei (accessed April 18, 2022).

[41] Onvani S Haghighatdoost F Surkan PJ Larijani B Azadbakht L. Adherence to the Healthy Eating Index and Alternative Healthy Eating Index dietary patterns and mortality from all causes, cardiovascular disease and cancer: a meta-analysis of observational studies. J Hum Nutr Dietet. (2017) 30:216–26. 10.1111/jhn.12415 27620213

[42] Bowman SA Lino M Gerrior SA Basiotis PP editors. The Healthy Eating Index: 1994-96. Washington, DC: Department of Agriculture Center for Nutritional Policy and Promotion (1998), p. 29. (CNPP-5).

[43] 2009 HHS Poverty Guidelines. ASPE. Available online: https://aspe.hhs.gov/2009-hhs-poverty-guidelines (accessed December 17, 2021).

[44] Gleeson M Bishop NC Stensel DJ Lindley MR Mastana SS Nimmo MA. The anti-inflammatory effects of exercise: mechanisms and implications for the prevention and treatment of disease. Nat Rev Immunol. (2011) 11:607–15. 10.1038/nri3041 21818123

[45] Harrison AL Taylor NF Shields N Frawley HC. Attitudes, barriers and enablers to physical activity in pregnant women: a systematic review. J Physiother. (2018) 64:24–32. 10.1016/j.jphys.2017.11.012 29289592

[46] Melbye H Hvidsten D Holm A Nordbø SA Brox J. The course of C-reactive protein response in untreated upper respiratory tract infection. Br J Gen Pract. (2004) 54:653–8. 10.22004/ag.econ.257277 15353049 PMC1326064

[47] Korn EL Graubard BI Midthune D. Time-to-event analysis of longitudinal follow-up of a survey: choice of the time-scale. Am J Epidemiol. (1997) 145:72–80. 10.1093/oxfordjournals.aje.a009034 8982025

[48] Kushi LH Doyle C McCullough M Rock CL Demark-Wahnefried W Bandera EV. American Cancer Society Guidelines on nutrition and physical activity for cancer prevention: reducing the risk of cancer with healthy food choices and physical activity. CA Cancer J Clin. (2012) 62:30–67. 10.3322/caac.20140 22237782

[49] Kohler LN Garcia DO Harris RB Oren E Roe DJ Jacobs ET. Adherence to diet and physical activity cancer prevention guidelines and cancer outcomes: a systematic review. Cancer Epidemiol Biomarkers Prev. (2016) 25:1018–28. 10.1158/1055-9965.EPI-16-0121 27340121 PMC4940193

[50] Meyerhardt JA Giovannucci EL Holmes MD Chan AT Chan JA Colditz GA. Physical activity and survival after colorectal cancer diagnosis. J Clin Oncol. (2006) 24:3527–34. 10.1200/JCO.2006.06.0855 16822844

[51] Cancer of the Breast (Female) - Cancer Stat Facts. SEER. Available online at: https://seer.cancer.gov/statfacts/html/breast.html (accessed June 10, 2022).

[52] Cancer of the Colon and Rectum - Cancer Stat Facts. SEER. Available online at: https://seer.cancer.gov/statfacts/html/colorect.html (accessed June 10, 2022).

[53] World Cancer Research Fund/American Institute for Cancer Research. Diet, Nutrition, Physical Activity and Colorectal Cancer. (2017). p. 111.

[54] Ballard-Barbash R Friedenreich CM Courneya KS Siddiqi SM McTiernan A Alfano CM. Physical activity, biomarkers, and disease outcomes in cancer survivors: a systematic review. J Natl Cancer Inst. (2012) 104:815–40. 10.1093/jnci/djs207 22570317 PMC3465697

[55] Kruijsen-Jaarsma M Révész D Bierings MB Buffart LM Takken T. Effects of exercise on immune function in patients with cancer: a systematic review. Exerc Immunol Rev. (2013) 19:120–43. 23977724

[56] Brown JC Winters-Stone K Lee A Schmitz KH. Cancer, physical activity, and exercise. Compr Physiol. (2012) 2:2775–809. 10.1002/cphy.c120005 23720265 PMC4122430

[57] Qiu J Yu Y Fu Y Ye F Xie X Lu W. Preoperative plasma fibrinogen, platelet count and prognosis in epithelial ovarian cancer. J Obstet Gynaecol Res. (2012) 38:651–7. 10.1111/j.1447-0756.2011.01780.x 22413879

[58] Crusz SM Balkwill FR. Inflammation and cancer: advances and new agents. Nat Rev Clin Oncol. (2015) 12:584–96. 10.1038/nrclinonc.2015.105 26122183

[59] Coussens LM Werb Z. Inflammation and cancer. Nature. (2002) 420:860–7. 10.1038/nature01322 12490959 PMC2803035

[60] Lichtman SW Pisarska K Berman ER Pestone M Dowling H Offenbacher E. Discrepancy between self-reported and actual caloric intake and exercise in obese subjects. N Engl J Med. (1992) 327:1893–8. 10.1056/NEJM199212313272701 1454084

[61] Bandini LG Schoeller DA Cyr HN Dietz WH. Validity of reported energy intake in obese and nonobese adolescents. Am J Clin Nutr. (1990) 52:421–5. 10.1093/ajcn/52.3.421 2393004

[62] Mertz W Tsui JC Judd JT Reiser S Hallfrisch J Morris ER. What are people really eating? The relation between energy intake derived from estimated diet records and intake determined to maintain body weight. Am J Clin Nutr. (1991) 54:291–5. 10.1093/ajcn/54.2.291 1858692

[63] Davies NJ Batehup L Thomas R. The role of diet and physical activity in breast, colorectal, and prostate cancer survivorship: a review of the literature. Br J Cancer. (2011) 105:S52–73. 10.1038/bjc.2011.423 22048034 PMC3251953

[64] Subar AF Freedman LS Tooze JA Kirkpatrick SI Boushey C Neuhouser ML. Addressing current criticism regarding the value of self-report dietary data12. J Nutr. (2015) 145:2639–45. 10.3945/jn.115.219634 26468491 PMC4656907

[65] Ahluwalia N Dwyer J Terry A Moshfegh A Johnson C. Update on NHANES dietary data: focus on collection, release, analytical considerations, and uses to inform public policy. Adv Nutr. (2016) 7:121–34. 10.3945/an.115.009258 26773020 PMC4717880

[66] NHANES III (1988-1994). Available online at: https://wwwn.cdc.gov/nchs/nhanes/nhanes3/default.aspx (accessed December 11, 2021).

[67] Pate RR Pratt M Blair SN Haskell WL Macera CA Bouchard C. Physical activity and public health. A recommendation from the Centers for Disease Control and Prevention and the American College of Sports Medicine. JAMA. (1995) 273:402–7. 10.1001/jama.1995.03520290054029 7823386

[68] Holmes MD Murin S Chen WY Kroenke CH Spiegelman D Colditz GA. Smoking and survival after breast cancer diagnosis. Int J Cancer. (2007) 120:2672–7. 10.1002/ijc.22575 17278091

